# The potential adverse effect of energy drinks on executive functions in early adolescence

**DOI:** 10.3389/fpsyg.2014.00457

**Published:** 2014-05-20

**Authors:** Tamara Van Batenburg-Eddes, Nikki C. Lee, Wouter D. Weeda, Lydia Krabbendam, Mariette Huizinga

**Affiliations:** ^1^Department of Educational Neuroscience and LEARN! Research Institute, Faculty of Psychology and Education, VU University AmsterdamAmsterdam, Netherlands; ^2^Department of Clinical Neuropsychology, Faculty of Psychology and Education, VU University AmsterdamAmsterdam, Netherlands

**Keywords:** energy drink use, puberty, executive functions, cognitive functioning, pubertal brain development

## Abstract

**Introduction:** Manufacturers of energy drinks (EDs) claim their products improve cognitive performance. Young adolescents are in a critical developmental phase. The impact of ED intake on their development is not yet clear. Therefore, we studied the associations of both caffeine intake and ED consumption with executive functions (EFs), and the role of pubertal status and sleeping problems.

**Methods:** A sample of 509 participants (mean age: 13.1 years, *SD* 0.85; age range: 11–16 years) participated in the study. The level of pubertal development was classified in five pubertal status categories. Participants were asked to report their caffeine (for example coffee) and ED consumption for each day of the week. In addition, they indicated sleep quality by reporting problems falling asleep or waking up and/or interrupted sleep. EFs were assessed by self- and parent reports of the Behavior Rating Inventory of Executive Function (BRIEF).

**Results:** Consuming on average one or more ED(s) a day was associated with more problems in self-reported behavior regulation and metacognition, and with more problems in parent-reported metacognition. Only high caffeine consumption (two or more cups a day) was associated with parent-reported problems with metacognition. The sum of caffeine and ED use was associated with a higher amount of problems with self-reported metacognition and parent reported behavior regulation. The effect estimates for the association between caffeine and ED use combined and EFs did not exceed those of EDs or caffeine separately. Adjusting for pubertal status, gender, educational level, number of sleeping problems and hours of sleep did not change the effect estimates substantially.

**Conclusion:** The observed associations between ED consumption and EFs suggest that regular consumption of EDs—even in moderate amounts—may have a negative impact on daily life behaviors related to EF in young adolescents.

## Introduction

Since the introduction of “Red Bull” in Austria in 1987, the consumption of caffeinated drinks has grown immensely (Reissig et al., 2009). Most of these so-called “energy drinks” (EDs) are marketed directly to children and adolescents and, at the same time, the use of these drinks within this population has risen exponentially (Bramstedt, 2007). According to Seifert et al. (2011), 30 to 50% of adolescents and young adults reported in surveys to consume EDs. A recent European study showed that the prevalence of “high chronic” ED users (i.e., respondents who regularly consumed ED “4–5 days a week” or more), is highest among Dutch adolescents (27%) compared to the prevalence of “high chronic ED use” in the other participating European countries (range 7–19%; Zucconi et al., 2013). These observations suggest that prolonged and habitual use of EDs is present in a substantial group of young adolescents.

Caffeine is the most important ingredient of EDs and is typically used for its arousing effect on the central nervous system (Seifert et al., 2011). Although caffeine is a psychoactive substance, it is considered safe by the Food and Drug Administration (Temple, 2009). However, excessive use of caffeine can have detrimental health effects (Seifert et al., 2011).

In the US, EDs are classified as dietary supplements and therefore the amount of caffeine content in these drinks is not regulated [US Food and Drug Administration. Q & A on Dietary Supplements (http://www.fda.gov/Food/DietarySupplements/QADietarySupplements/default.htm)]. In the Netherlands, however, by law the maximum allowed caffeine content is 350 mg/l, which is determined by the Netherlands Food and Consumer Product Safety Authority (http://www.vwa.nl/actueel/bestanden/bestand/42527). If the caffeine content exceeds 150 mg/l, manufactures are obliged to print “high caffeine content” on their products. Furthermore, the Dutch Food Foundation advises young adolescents between the age of 13 and 18 years to consume at most one can of ED (250 ml) a day (Netherlands Food and Consumer Product Safety Authority; Stichting Voedingscentrum Nederland, 2013).

Manufacturers of EDs claim their products improve physical and cognitive performance. The direct short-term positive effect on cognitive performance is still controversial, but when it is found it is often attributed to caffeine. The effect of caffeine on particular aspects of cognitive functioning has been observed in numerous well-controlled studies in a range of populations (Lieberman, 2001). In addition, Wesnes et al. (2013) conclude that many studies have detected improvements of cognitive functioning or alertness after ingesting caffeine or EDs. However, they also point out that most studies have investigated the short-term effects 1–2 h after ingestion. Wesnes et al. (2013) studied the effect of a specific ED 6 h after ingestion. They found a sustained effect of this specific ED in partially sleep deprived participants on four out of six cognitive performance tasks after consuming this ED compared to the placebo group (Wesnes et al., 2013). In contrast, Curry and Stasio (2009) investigated the effects of EDs alone and combined with alcohol on neuropsychological functioning. In the ED only group, they found a trend toward improved attention and no overall improvement in neuropsychological functioning from pre-to post-test. Furthermore, as little is known about the effects of EDs on cognitive functioning in young persons, Wilhelm et al. (2013) studied young adolescents aged 15 to 18 years. In a quasi-experimental design comparing three groups, no significant differences were observed between the groups that could be ascribed to the effect of ED on measures of cognitive functioning such as attention, learning ability and vocabulary. Nevertheless, all these studies focused on relatively short-term effects of EDs (i.e., effects on cognitive functioning within a certain time after consuming the ED). The effect of prolonged and habitual use of EDs on more long-term every day cognitive functioning has, to the best of our knowledge, not been studied in young adolescents.

The long-term effects of caffeine consumption and ED use during adolescence may have consequences for adolescent development. Adolescence is a period characterized by continued structural and functional brain development, triggered by the hormonal changes at the onset of puberty (Giedd, 2004; Gogtay et al., 2004; Paus, 2013). The prefrontal cortex, one of the areas of the brain that shows the greatest development during this period, contains areas involved in a variety of cognitive abilities, including executive functions. These are vital for an individual to be able to control and reflect on their behavior, and to be able to behave in a goal-directed manner. Executive functions continue to develop and improve steadily throughout adolescence and into adulthood (Huizinga et al., 2006). As young adolescents are in a critical developmental phase, this may make them in particularly vulnerable to the potential negative effects of caffeinated drinks.

Regular caffeine consumption has been related to numerous potential adverse outcomes, such as cardiovascular effects, caloric intake, diabetes and problems related to sleep (Roehrs and Roth, 2008; Seifert et al., 2011). The relation between regular caffeine consumption and disrupted sleep and increased daytime sleepiness seems to be well-established. Although adolescents seem to consume caffeinated drinks (including coffee) to a lesser extent than adults, in this age group caffeine use is also associated with sleeping problems and daytime sleepiness (see for a review: Roehrs and Roth, 2008). Furthermore, sleep seems to be particularly important during periods of brain maturation, such as adolescence (Dahl and Lewin, 2002). In addition, sleep deprivation during adolescence is related to a wide range of behavioral deficits, such as attention problems, oppositionality/irritability, behavior regulation problems, and reduced metacognitive skills (Beebe et al., 2008; O'Brien, 2009; Jackson et al., 2013).

The main goal of the present study was to investigate, in a sample of young adolescents, the associations between caffeine intake and ED consumption, and behavioral executive function and metacognition. We examined the effects of these caffeine and EDs individually and cumulatively. Because of the previously reported relations between caffeine use and sleep, and between sleep and cognitive functioning, indicators of sleeping problems were included in the current study to investigate their potential mediating effect in the relation between caffeine intake and ED consumption with cognitive functioning.

## Materials and methods

### Study population

This study included 564 young adolescents (*M* age 13.10 years, *SD* = 0.85; age range: 11–16 years; 244 females). Participants were recruited through four regular schools in urban and suburban areas in the Netherlands (see Table 1 for sample characteristics). This study is part of a longitudinal project focusing on young adolescents' socio-emotional and cognitive development. Participants completed multiple questionnaires and cognitive tasks, including a questionnaire on executive functioning (self-report and parent report); 509 participants (and 317 of their parents) filled in this questionnaire and were therefore included in the analysis. The total sample thus consisted of 509 participants. Informed consent was obtained, and the study was approved by the Ethical Committee of the Faculty of Behavioral and Social Sciences of the University of Amsterdam. Participants did not receive credit individually, but received a voucher for an excursion together with participating classmates.

**Table 1:** **Sample characteristics**.

	**Sample of *n* = 509 pupils**
Gender, boys, %	52
Age at assessment in years, mean (*SD*)	13.10 (0.85)
Pubertal status category, %	
Prepubertal	8
Early puberty	23
Midpubertal	37
Late puberty	29
Postpuberty	2
Educational track, %	
Primary school	22
Pre-vocational secondary education	32
Pre-vocational/senior general secondary education	8
Senior general secondary education	10
Senior general secondary/pre-university education	16
Pre-university education	11
Caffeine, %	
<1 per day	72
≥1–2 each day	17
≥2 each day	11
EDs, %	
<1 per day	94
≥1 each day	6
Caffeine and EDs, %	
<1 per day	65
≥1–2 each day	21
≥2 each day	14
Problems falling asleep, yes, %	23
Problems staying asleep, yes, %	6
Problems waking up, yes, %	23
Total of sleeping problems, mean (*SD*)	0.51 (0.79)
Hours of sleep, mean (*SD*)	8.83 (1.26)

When comparing the included sample of parents (*n* = 317) to the sample of parents that was excluded because of missing parent reported data on executive functioning (*n* = 192), we found no statistically significant differences in gender, pubertal status category, caffeine or energy drink intake, or on the variables regarding sleeping, age at assessment, and BRIEF scores.

### Measures

#### Caffeine and energy drink intake

Caffeine and energy drink intake were measured separately by asking the participants how often, during a normal/average week they consumed caffeine (coffee or cola), and how often they consumed energy drinks (Red Bull, Xii etc.; Graham et al., 1984; Ames et al., 2007). For each day of the week, participants indicated the number of consumed cups, cans, or glasses. These numbers were summed for caffeine use and ED intake, and for each were divided by 7 to derive the average number of consumptions of caffeine and the average number of EDs per day. As caffeine use and ED intake were correlated (Pearson *r* = 0.36, *p* < 0.001), we also calculated the total number of caffeine and ED consumptions, by summing the number of caffeine and ED consumptions. Scores were divided by 7, yielding the average number of caffeine containing drinks consumed per day.

#### Executive functions

Executive functions were assessed with the self-report and parent-report of the Dutch Behavior Rating Inventory of Executive Function (BRIEF: Gioia et al., 2000, 2002; Smidts and Huizinga, 2009). In contrast to experiments that enable researchers to measure specific cognitive functions as the settings can be controlled to a large degree, self-reports and parents measure executive functioning in real life settings, and thus offer increased ecological validity. The self- and parent reports of the BRIEF consists of 70 items, whereas the parent-report of the BRIEF consists of 75 items. Each item pertains to specific everyday behavior, relevant to executive functioning. Children and their parents were asked to indicate how often they or their child displayed a given problem behavior in the past 6 months. Scoring options were “1 = Never,” “2 = Sometimes,” or “3 = Often.” Higher scores indicate more problems. Self-report items are categorized into eight clinical scales: Inhibit, Shift, Emotional Control, Monitor, Working Memory, Plan/Organize, Organization of Materials, and Persistence. In the current sample, the range of alpha's for internal consistency for the clinical subscales was 0.71 and 0.84. Parent-report items are also categorized into eight clinical scales but without the Persistence scale and with an additional scale measuring Initiative. In the current sample, the range of alpha's for internal consistency for the clinical subscales was 0.80 and 0.89. To calculate the clinical scale scores, the appropriate item scores were summed and divided by the number of items in each scale.

Two indices—the Behavior Regulation Index (BRI) and the Metacognition Index (MI)—can be formed by combining scales. The BRI represents the ability to shift cognitive set and modulate behavior and emotions, whereas the MI represents the ability to plan, organize, initiate, and hold information in mind for future-oriented problem solving. The mean across the appropriate clinical scale scores was calculated to yield the BRI and MI indices. In the current sample, the range of alpha's for internal consistency for the self-reported and parent reported indices was 0.91 and 0.96. In addition, raw scores were transformed into T-scores based on the Dutch norm population (Huizinga and Smidts, 2012).

#### Covariates

Pubertal status category was determined by means of the self-report Pubertal Development Scale, which was developed by Carskadon and Acebo (1993) as an adaptation of the interview-based puberty-rating scale by Petersen et al. (1988). The scale measures pubertal status using a 5-point scale to rate five questions indexing physical development. Both boys and girls are asked to rate their development with regards to growth in height, body hair growth, skin changes. For boys there are additional questions about voice change and facial hair growth and for girls there are additional questions about breast development and menarche. Answers are rated on a 4-point scale, with 1 indicating no development, and 4 indicating that development is finished, and 5 indicating “I don't know” or a missing value. The question about menarche was coded dichotomously (1 = premenarcheal, 4 = postmenarcheal). An individual's level of development was classified in terms of five pubertal status categories: prepubertal, early pubertal, mid-pubertal, late pubertal, and postpubertal. For boys, the assignment was made on the basis of reported level of body hair growth, facial hair growth, and voice change. Girls were assigned on the basis of reported level of body hair growth and breast development and whether or not a girl reported having experienced menarche (Carskadon and Acebo, 1993).

Participants answered three questions about sleep problems: (1) problems falling asleep, (2) problems staying asleep, and (3) problems waking up in the morning. Answering categories were “yes” (1), or “no” (0). The answers across the three questions about sleeping were summed yielding “total number of sleeping problems”.

The demographic variables gender and educational track were also measured using a questionnaire.

### Statistical analyses

Linear regression analysis was used to assess the associations between caffeine and/or ED intake and EF. In these analyses, caffeine and/or ED intake variables were the independent variables, all BRIEF measures were used as dependent variables. For each pair of independent and dependent variable, a separate linear regression analysis was done. Indices of behavioral regulation and metacognition were regarded as the main overall outcome measures, as these are summary measures of the clinical subscales. The associations between each independent variable and each clinical subscale measure were inspected to determine which association(s) contributed to the overall association between determinant and index. Caffeine and/or ED intake variables were dummy-coded with on average using less than one consumption of these drinks a day was used as the reference category. First, the associations between caffeine and/or ED intake and EF were adjusted for gender and pubertal status category, as gender was associated with parent reported MI [F_(1,315)_ = 8.63, *p* = 0.004] and pubertal status category was associated with self-reported MI [F_(4,438)_ = 3.08, *p* = 0.016]. Also, educational level was added as a potential confounder, as it was weakly associated with self-reported metacognition (*r* = 0.16, *p* < 0.001). Next, we added total number of sleeping problems and hours of sleep as potential mediating factors in the associations between caffeine and/or ED intake and EFs. The effect of sleeping problems and hours of sleep on the associations under study was small. Sleeping problems and hours of sleep were still included in our analyses because of their well-known associations with caffeine use and/or ED intake and EFs, but regression models excluding sleeping problems and hours of sleep are not presented separately. Thus, the final models were adjusted for gender, pubertal status category, educational track, sleeping problems and hours of sleep. We adjusted for multiple testing as follows. First, the *p*-value of the associations between caffeine use and/or ED intake and the overall indices was set 0.025. For this set of associations, we ran four tests. As the indices are related to each other (Pearson *r*_*SR BRI & MI*_ = 0.75, *p* < 0.001; Pearson *r*_*PR BRI & MI*_ = 0.65, *p* < 0.001) and the independent variables, caffeine use and ED intake, are also related (Pearson *r* = 0.36, *p* < 0.001), the desirable *p*-value 0.05 was divided by four and then multiplied by two to compensate for the correlational structure (Bender and Lange, 2001). Next, we set the *p*-value for the analyses using the clinical subscales as outcome measures at = 0.01 (i.e., 0.05 divided by five which is the maximum number of clinical subscales for the indices).

The main analyses assessing the associations between caffeine and ED intake and EFs were repeated using *T*-scores of the BRIEF scales and indices. Results were consistent with those based on the raw scores and are therefore not reported.

## Results

Sample characteristics are displayed in Table 1. Most of the participants were in the early pubertal stage (23%), midpubertal stage (37%), or late pubertal stage (29%). Eight percent of participants was in the prepubertal stage, whereas only 2% was in the postpubertal stage. 11% reported to drink, during normal weeks, on average at least two caffeine containing drinks a day such as coffee or cola (excluding energy drinks). Six percent reported to consume on average at least one energy drink a day. Problems with falling asleep and waking up were reported most often (23%). These characteristics were highly similar in the sample for which we also had parent reports of EFs.

The adjusted associations between caffeine consumption and/or ED intake and self-reported behavioral executive function and metacognition are shown in Tables 2A,B. Consuming on average one ED or more a day was associated with the BRI (B 0.14, 95% CI 0.03; 0.24, *p* = 0.012), indicating more problems with self-reported behavior regulation. This association was due to the subscales measuring Inhibition and Monitor.

**Table 2A:** **Associations between caffeine and energy drink consumption and self-reported behavioral executive functioning (***n*** = 509)**.

	**Inhibition**		**Emotional control**		**Monitor**		**Shift**		**BRI^#^**	
	***B* (95% CI)**	***p***	***B* (95% CI)**	***p***	***B* (95% CI)**	***p***	***B* (95% CI)**	***p***	***B* (95% CI)**	***p***
**CAFFEINE**										
<1 per day	Reference		Reference		Reference		Reference		Reference	
≥1–2 each day	0.06 (−0.02; 0.14)	0.13	−0.04 (−0.11; 0.04)	0.32	0.08 (−0.03; 0.18)	0.15	−0.03 (−0.10; 0.05)	0.50	0.02 (−0.05; 0.09)	0.58
≥2 each day	0.11 (0.01; 0.21)	0.05	0.01 (−0.08; 0.10)	0.75	0.04 (−0.08; 0.17)	0.49	0.01 (−0.09; 0.10)	0.94	0.04 (−0.04; 0.12)	0.33
**EDs**										
<1 per day	Reference		Reference		Reference		Reference		Reference	
≥1 each day	0.18 (0.05; 0.31)	0.008	0.07 (−0.05; 0.18)	0.28	0.22 (0.05; 0.38)	0.009	0.09 (−0.04; 0.21)	0.17	0.14 (0.03; 0.24)	0.012
**CAFFEINE AND EDs**										
<1 per day	Reference		Reference		Reference		Reference		Reference	
≥1–2 each day	0.09 (0.02; 0.17)	0.02	0.02 (−0.05; 0.08)	0.67	0.05 (−0.05; 0.14)	0.36	−0.02 (−0.09; 0.05)	0.59	0.03 (−0.03; 0.10)	0.30
≥2 each day	0.14 (0.05; 0.23)	0.05	0.02 (−0.06; 0.10)	0.66	0.08 (−0.03; 0.20)	0.15	0.01 (−0.08; 0.09)	0.90	0.06 (−0.01; 0.14)	0.10

Linear regression models adjusted for pubertal status, gender, educational track, number of sleeping problems and hours of sleep. Caffeine, EDs, and Caffeine and EDs represent the independent dummy-coded variables in which <1 consumption on average per day represents the reference category; self-reported behavioral executive functioning measures are the outcome variables. B, the estimate of increase in self-reported behavioral executive functioning score compared to the reference category; CI, Confidence Interval;

# *BRI, Behavior Regulation Index*.

**Table 2B:** **Associations between caffeine and energy drink consumption and self-reported metacognition (***n*** = 509)**.

	**Working memory**		**Planning and organizing**		**Organization of material**		**Persistence**		**MI^#^**	
	***B* (95% CI)**	***p***	***B* (95% CI)**	***p***	***B* (95% CI)**	***p***	***B* (95% CI)**	***p***	***B* (95% CI)**	***p***
**CAFFEINE**										
<1 per day	Reference		Reference		Reference		Reference		Reference	
≥1–2 each day	0.01 (−0.08; 0.09)	0.88	−0.01 (−0.09; 0.07)	0.79	0.01 (−0.08; 0.11)	0.80	−0.02 (−0.11; 0.06)	0.58	0.00 (−0.08; 0.07)	0.91
≥2 each day	0.05 (−0.06; 0.15)	0.38	0.06 (−0.04; 0.15)	0.27	0.11 (−0.01; 0.23)	0.07	0.07 (−0.03; 0.18)	0.18	0.07 (−0.02; 0.16)	0.12
**EDs**										
<1 per day	Reference		Reference		Reference		Reference		Reference	
≥1 each day	0.19 (0.06; 0.33)	0.006	0.15 (0.03; 0.28)	0.017	0.20 (0.05; 0.35)	0.011	0.15 (0.01; 0.29)	0.031	0.17 (0.06; 0.29)	0.003
**CAFFEINE AND EDs**										
<1 per day	Reference		Reference		Reference		Reference		Reference	
≥1–2 each day	−0.01 (−0.09; 0.07)	0.82	0.00 (−0.08; 0.07)	0.99	0.04 (−0.05; 0.13)	0.39	−0.01 (−0.09; 0.07)	0.80	0.01 (−0.06; 0.07)	0.89
≥2 each day	0.10 (0.01; 0.19)	0.05	0.06 (−0.03; 0.15)	0.18	0.13 (0.03; 0.24)	0.015	0.09 (−0.01; 0.18)	0.08	0.09 (0.01; 0.17)	0.023

Linear regression models adjusted for pubertal status, gender, educational track, number of sleeping problems and hours of sleep. Caffeine, EDs, and caffeine and EDs represent the independent dummy-coded variables in which <1 consumption on average per day represents the reference category; self-reported metacognition measures are the outcome variables. B, the estimate of increase in self-reported metacognition score compared to the reference category; CI, Confidence Interval;

# *MI, Metacognition Index*.

Participants who consumed at least one ED a day had higher scores on the MI (B 0.17, 95% CI 0.06; 0.29, *p* = 0.003). ED intake was associated with the metacognitive clinical subscales measuring Working Memory and Organization of Material. Participants who drank at least two consumptions of caffeine or ED had on average higher scores on the MI (B 0.09, 95% CI 0.01; 0.17, *p* = 0.023), indicating more problems with metacognitive skills. The effect estimate for this association was smaller compared to the effect estimate for the association between ED use and the MI. As the effect estimate of the sum of caffeine and ED use (B 0.09) falls within the CI of the association between ED use and the MI (95% CI 0.06;0.29), it is unlikely that there is a significant difference between the two associations. Furthermore, if there was any “cumulative effect,” we expected the effect estimate of combined caffeine and ED use to be higher and not smaller than the one for the association between EDs and the MI. Finally, consuming at least one ED a day was related to more problems with behavior regulation and metacognition, whereas caffeine consumption was not related to any of the self-reported outcomes.

The adjusted associations between caffeine consumption and ED intake and parent reported behavioral executive function and metacognition are presented in Tables 3A,B. Only, consuming on average one or two caffeine containing drinks or EDs was associated with higher scores on the parent reported BRI (B 0.12, 95% CI 0.04; 0.20, *p* = 0.005), which was due to the association between caffeine and ED consumption and Inhibition. There were no statistically significant associations between caffeine use and/or EDs and parent reported metacognitive skills.

**Table 3A:** **Associations between caffeine and energy drink consumption and parent reported behavioral executive functioning (*n* = 319)**.

	**Inhibition**		**Emotional control**		**Shift**		**BRI^−^^#^**	
	***B* (95% CI)**	***p***	***B* (95% CI)**	***p***	***B* (95% CI)**	***p***	***B* (95% CI)**	***p***
**CAFFEINE**								
<1 per day	Reference		Reference		Reference		Reference	
≥1–2 each day	0.11 (0.01; 0.21)	0.028	0.07 (−0.05; 0.18)	0.24	−0.02 (−0.12; 0.09)	0.74	0.05 (−0.04; 0.14)	0.24
≥2 each day	0.09 (−0.03; 0.20)	0.16	0.03 (−0.11; 0.17)	0.67	0.01 (−0.13; 0.14)	0.93	0.04 (−0.07; 0.15)	0.47
**EDs**								
<1 per day	Reference		Reference		Reference		Reference	
≥1 each day	0.04 (−0.13; 0.21)	0.64	0.00 (−0.21; 0.20)	0.96	−0.03 (−0.22; 0.16)	0.77	0.03 (−0.15; 0.16)	0.97
**CAFFEINE AND EDs**								
<1 per day	Reference		Reference		Reference		Reference	
≥1–2 each day	0.15 (0.06; 0.24)	0.002	0.13 (0.02; 0.24)	0.018	0.08 (−0.02; 0.18)	0.12	0.12 (0.04; 0.20)	0.005
≥2 each day	0.08 (−0.03; 0.18)	0.18	0.02 (−0.11; 0.15)	0.74	0.02 (−0.10; 0.14)	0.75	0.04 (−0.06; 0.14)	0.45

Linear regression models adjusted for pubertal status category, gender, educational track, number of sleeping problems and hours of sleep. Caffeine, EDs, and Caffeine and EDs represent the independent dummy-coded variables in which <1 consumption on average per day represents the reference category; parent reported behavioral executive functioning measures are the outcome variables. B, the estimate of increase in parent reported behavioral executive functioning score compared to the reference category; CI, Confidence Interval;

# *BRI, Behavior Regulation Index*.

**Table 3B:** **Associations between caffeine and energy drink consumption and parent-reported metacognition (*n* = 319)**.

	**Initiative**		**Working memory**		**Planning and organizing**		**Organization of material**		**Monitor**		**MI ^#^**	
	***B* (95% CI)**	***p***	***B* (95% CI)**	***p***	***B* (95% CI)**	***p***	***B* (95% CI)**	***p***	***B* (95% CI)**	***p***	***B* (95% CI)**	***p***
**CAFFEINE**												
<1 per day	Reference		Reference		Reference		Reference		Reference		Reference	
≥1–2 each day	0.04 (−0.09; 0.15)	0.57	0.02 (−0.11; 0.15)	0.79	0.03 (−0.08; 0.14)	0.63	0.04 (−0.11; 0.20)	0.58	0.07 (−0.05; 0.19)	0.24	0.04 (−0.07; 0.14)	0.47
≥2 each day	0.12 (−0.03; 0.27)	0.11	0.09 (−0.07; 0.25)	0.26	0.12 (−0.02; 0.25)	0.09	0.19 (0.00; 0.38)	0.05	0.12 (−0.03; 0.27)	0.11	0.13 (0.00; 0.26)	0.05
**EDs**												
<1 per day	Reference		Reference		Reference		Reference		Reference		Reference	
≥1 each day	0.18 (−0.04; 0.39)	0.10	0.15 (−0.08; 0.37)	0.21	0.22 (0.03; 0.41)	0.026	0.14 (−0.13; 0.41)	0.30	0.22 (0.01; 0.43)	0.039	0.18 (0.00; 0.37)	0.05
**CAFFEINE AND EDs**												
<1 per day	Reference		Reference		Reference		Reference		Reference		Reference	
≥1–2 each day	0.02 (−0.10; 0.13)	0.78	0.04 (−0.08; 0.16)	0.52	0.07 (−0.03; 0.18)	0.17	0.08 (−0.07; 0.23)	0.28	0.15 (0.03; 0.26)	0.013	0.07 (−0.03; 0.17)	0.16
≥2 each day	0.12 (−0.02; 0.25)	0.09	0.08 (−0.07; 0.22)	31	0.12 (−0.01; 0.24)	0.07	0.12 (−0.05; 0.30)	0.16	0.10 (−0.04; 0.24)	0.14	0.11 (−0.01; 0.23)	0.08

Linear regression models adjusted for pubertal status category, gender, educational track, number of sleeping problems and hours of sleep. Caffeine, EDs, and Caffeine and EDs represent the independent dummy-coded variables in which <1 consumption on average per day represents the reference category; parent reported metacognition measures are the outcome variables. B, the estimate of increase in parent reported metacognition score compared to the reference category; CI, Confidence Interval;

# *MI, Metacognition Index*.

### Additional analyses

Supplementary Tables 1 and 2 show how the magnitude of associations between caffeine and/or ED use and the indices changes by adding the potential confounders. “Models 1” show the unadjusted models. Adding gender, pubertal status and educational track, only slightly reduced the effect estimates.

## Discussion

The present study showed that during early adolescence consuming on average at least one ED a day was associated with more problems regarding behavior regulation and metacognition. Although caffeine use in the current sample was higher than ED consumption, we found no statistical significant associations between caffeine use and EFs. The sum of caffeine and ED consumptions was associated with self-reported problems with metacognition and with parent reported behavior regulation, but the effect estimates of these associations did not seem to be statistically different from those of the associations between ED and EFs. Both effect estimates of combined use of caffeine and ED use fell within the confidence interval of the effect estimates of the association between ED use and EFs.

EDs, according to their manufacturers, enhance physical and, relevant to the current study, cognitive performance. Scientific studies investigating the effect of ED intake on cognitive performances either found improvement on cognitive tasks after consuming EDs (e.g., Lieberman, 2001; Wesnes et al., 2013) or found no evidence of an effect (e.g., Curry and Stasio, 2009; Wilhelm et al., 2013). In contrast, we found that ED intake was associated with an increased amount of problems with behavior regulation and metacognition. There are several potential explanations for the discrepancies in findings. First, fundamental differences in methods of assessment may have contributed to the differences between our findings and those of earlier studies. Previously conducted research focused on direct short-term effects of ED use on neurocognitive functioning assessed in experimental settings. ED use in this type of settings is by definition occasional use. We investigated the potential effect of habitual ED use on long-term cognitive functioning in daily life situations, measured by self-reports and parent reports. Although experiments enable researchers to precisely control the settings to measure certain cognitive functions, self-reports and parent reports give more insight in problems with executive functions in real life settings. Self-reports and parent reports, such as the BRIEF, measure executive function problems in a real-world setting and thus offer a higher ecological validity compared to laboratory based measures. In general, no or low correlations are reported between BRIEF measurements and performance-based measures (see for an overview in the literature: Huizinga and Smidts, 2011), which further illustrates that questionnaires and experimentally based measures are likely to tap different constructs (see also Toplak et al., 2013). Second, most previously conducted research studied the effects of EDs in older adolescents (Curry and Stasio, 2009; Wilhelm et al., 2013) or in samples with a broad age range (Wesnes et al., 2013). Our study focused specifically on young adolescents who are just entering their development toward adulthood, i.e., puberty. At the onset of puberty, the young adolescents' brain goes through a critical developmental phase, in which the maturation of the prefrontal brain areas plays a substantial role and has a large impact on a variety of cognitive functions. Our findings suggest that young adolescents who consume EDs on a regular basis perform worse in EFs than their non-using counterparts. Although the effects of caffeine consumption on brain development have not yet been examined, Temple (2009) hypothesizes that caffeine may alter normal brain development during critical developmental periods. This idea stems from animal models in which perinatal caffeine exposure had long lasting effects on brain function (For a review, see Temple, 2009). Third, it is possible that young adolescents that tend to consume caffeine and EDs may do so because of their - already - compromised EFs. EF may improve by caffeine and ED intake but may not be fully compensated in youngsters that use these drinks, i.e., in young adolescents who experience problems with EF. Finally, when interpreting the observed findings, it is important to take into account that 6% of our sample consumed on average at least one ED a day. Furthermore, the effect estimates of the associations between ED use and EFs were relatively small. These results may limit the impact of our findings.

Caffeine's stimulating effect on the central nervous system is well-established, and the capability of EDs to improve cognitive performances is often attributed to the caffeine they contain (Seifert et al., 2011). Therefore, we expected the associations between caffeine use and EFs vs. EDs and EFs to be similar. However, caffeine use was not associated with any of the outcome measures. Several explanations may underlie these findings. First, the quantity of caffeine in EDs can be substantially larger than in caffeine drinks. For example, the content of a regular cup of coffee usually varies between 125–250 ml, whereas cans of EDs vary between 250–500 ml. Second, in addition to caffeine, EDs contain high levels of sugar and smaller amounts of several other substances, such as vitamins, minerals, ginseng, taurine, inositol or other herbal extracts. In contrast to the idea that the effect of EDs is mainly due to the caffeine content, findings of other studies suggest that the combination of EDs' ingredients work synergistically (Scholey and Kennedy, 2004; Smit et al., 2004; Temple, 2009). These discrepancies in our findings, necessitate further research on EDs and ED components to determine their potential threats or benefits for health and performance.

Caffeine use and ED intake were moderately correlated. Therefore, we expected to find indications of a cumulative effect of combined use of caffeine and EDs. The effect estimate for the association between combined caffeine and ED use and parent reported BRI was, in absolute value, larger than the effect estimate for the association between EF and BRI, but fell within its confidence interval. The effect estimate for the association between combined caffeine and ED use and self-reported BRI was, in absolute value, even smaller than the effect estimate for the association between EDs and BRI, and also fell within its confidence interval. Therefore, it is implausible that the associations between the sum of caffeine consumptions and EDs are statistically different from the associations for caffeine and EDs separately. The moderate correlation between caffeine use and ED intake, indicating that few adolescents consumed both products, may have contributed to this finding.

### Strengths and limitations

This study has several strengths. First, this study is a first attempt to shed light on the potential long-term effects of ED intake on behavior or cognitive functioning in daily life situations, as previous research has focused on short-term direct effects of ED intake on cognitive functions. Second, in this study we investigated a large sample of specifically young adolescents entering or in puberty. We focused on this particular developmental phase as it can be determinative for later life functioning.

Several methodological limitations need to be discussed. First, participants reported the number of consumptions. Therefore, the exact amount of caffeine present in each consumption was unknown. Future research needs to focus on exact ED or caffeine intake by asking more detailed questions about the consumed brands and exact quantities of each consumption. Second, due to the cross sectional design of the study, we cannot ensure the proposed directions of associations. It is possible that caffeine and EDs influence EFs, but the opposite is also feasible. As was mentioned earlier, young adolescents that use caffeine and EDs on a regular basis, may have certain characteristics, for example, compromised EFs beforehand, which may have influenced their ED intake instead of vice versa.

### Implications and conclusions

Since the introduction of EDs, its market has grown immensely (Report Buyer, 2007). The prevalence of ED use has especially risen among children and adolescents (Reissig et al., 2009; Seifert et al., 2011; Zucconi et al., 2013). The fast rise in prevalence of ED use in this young population which is in a critical developmental phase is alarming, as there is a lack of research on the long term consequences of prolonged and regular use of these EDs on physical and cognitive health. Our findings suggest a possible negative effect of ED use on behavior regulation and metacognitive skills. However, it is important to note that in the current sample a relatively small group of young adolescents consumed on average EDs on a daily basis. Also, effect estimates were relatively small. We conducted a correlational study, therefore, no inferences can be made about causality or effects. Taken together, these results may limit the impact of our findings. Our findings do support the need for further more detailed research on the consequences of ED use in this vulnerable population. Future research on the possible negative effects of ED use on the more long-term EFs in daily life situations should focus on, for example on the exact amount of ED use, other sources of caffeine intake, and directions of associations.

## Author contributions

Tamara Van Batenburg-Eddes performed the analyses for the current study, wrote the concept drafts and approved the final version of the manuscript, and takes full responsibility for all aspects of the work in terms of accuracy and integrity.

Nikki C. Lee had a substantial contribution to the interpretation of the data, writing and revising the manuscript, and approved the final version of the manuscript. She agrees to be accountable for all aspects of the work in terms of accuracy and integrity.

Wouter D. Weeda had a substantial contribution to the design of the work, analyses and interpretation of the data. Wouter D. Weeda contributed to revising the manuscript and approves of the final version to be published and agrees to be accountable for all aspects of the work in terms of accuracy and integrity.

Lydia Krabbendam is co-designer of the study, reviewed the manuscript and approved the final version of it and agrees to be accountable for all aspects of the work in terms of accuracy and integrity.

Mariette Huizinga is guarantor and designer of the study, supervised the first author, wrote and reviewed the manuscript and approved the final version of it, and takes full responsibility for all aspects of the work in terms of accuracy and integrity.

### Conflict of interest statement

The authors declare that the research was conducted in the absence of any commercial or financial relationships that could be construed as a potential conflict of interest.
