# Supplementary material for: The potential adverse effect of energy drinks on executive functions in early adolescence
Source: Front Psychol. 2014 May 20;5:457. doi: 10.3389/fpsyg.2014.00457 (PMC4033167; doi:10.3389/fpsyg.2014.00457)
Supplement: Supplementary file 1 [file DataSheet1.PDF]

Supplementary table 1: Unadjusted associations between caffeine and energy drink consumption and self-reported indices of behavioral executive functioning and metacognition (n=509)

	**BRI**				**MI**			
	**Model 1**		**Model 2**		**Model 1**		**Model 2**	
	**B (95% CI)**	**p**	**B (95% CI)**	**p**	**B (95% CI)**	**p**	**B (95% CI)**	**p**
Caffeine								
< 1 per day	Reference		Reference		Reference		Reference	
≥1-2 each day	0.04 (-0.03; 0.11)	.26	0.03 (-0.03; 0.10)	.33	0.02 (-0.05; 0.10)	.53	0.02 (-0.06; 0.09)	.53
≥2 each day	0.07 (-0.01; 0.15)	.097	0.06 (-0.02; 0.15)	.16	0.11 (0.02; 0.20)	.017	0.10 (0.01; 0.19)	.034
EDs								
< 1 per day	Reference		Reference		Reference		Reference	
≥1 each day	0.15 (0.04; 0.26)	.013	0.14 (0.03; 0.25)	.011	0.18 (0.06; 0.30)	.003	0.19 (0.07; 0.31)	.002
Caffeine and EDs								
< 1 per day	Reference		Reference		Reference		Reference	
≥1-2 each day	0.06 (-0.01; 0.12)	.083	0.04 (-0.02; 0.11)	.19	0.03 (-0.04; 0.10)	.36	0.02 (-0.05; 0.09)	.36
≥2 each day	0.09 (0.02; 0.17)	.012	0.09 (0.02; 0.16)	.02	0.14 (0.06; 0.22)	.001	0.14 (0.05; 0.22)	.001

Linear regression models
Caffeine, EDs, and caffeine and EDs represent the independent dummy-codes variables in which <1 consumption on average per day represents the reference category; BI or MI measures are the outcome variables; for each pair of independent and dependent variable a separate linear regression model is presented
Model 1: Unadjusted linear regression models; Model 2: Linear regression models adjusted for gender, pubertal status, educational track
B = the estimate of increase in BRI or MI score compared to the reference category
CI=confidence interval
BRI=Behavior Regulation Index; MRI= Metacognition Index
