# Supplementary material for: The potential adverse effect of energy drinks on executive functions in early adolescence
Source: Front Psychol. 2014 May 20;5:457. doi: 10.3389/fpsyg.2014.00457 (PMC4033167; doi:10.3389/fpsyg.2014.00457)
Supplement: Supplementary file 2 [file DataSheet2.PDF]

Supplementary table 2: Unadjusted associations between caffeine and energy drink consumption and parent reported indices of behavioral executive functioning and metacognition (n=319)

	**BRI**				**MI**			
	**Model 1**		**Model 2**		**Model 1**		**Model 2**	
	**B (95% CI)**	**p**	**B (95% CI)**	**p**	**B (95% CI)**	**p**	**B (95% CI)**	**p**
Caffeine								
< 1 per day	Reference		Reference		Reference		Reference	
≥1-2 each day	0.05 (-0.04; 0.14)	.24	0.06 (-0.03; 0.15)	.22	0.07 (-0.04; 0.17)	.21	0.04 (-0.06; 0.15)	.44
≥2 each day	0.05 (-0.06; 0.15)	.41	0.05 (-0.06; 0.16)	.33	0.17 (0.05; 0.30)	.008	0.15 (0.02; 0.28)	.028
EDs								
< 1 per day	Reference		Reference		Reference		Reference	
≥1 each day	0.03 (-0.12; 0.19)	.69	0.01 (-0.15; 0.16)	.95	0.24 (0.05; 0.42)	.011	0.19 (0.00; 0.37)	.05
Caffeine and EDs								
< 1 per day	Reference		Reference		Reference		Reference	
≥1-2 each day	0.12 (0.04; 0.20)	.005	0.13 (0.05; 0.21)	.003	0.11 (0.01; 0.21)	.025	0.09 (-0.02; 0.19)	.09
≥2 each day	0.05 (-0.05; 0.14)	.35	0.05 (-0.05; 0.15)	.31	0.16 (0.04; 0.22)	.008	0.13 (0.07; 0.25)	.04

Linear regression models

Caffeine, EDs, and caffeine and EDs represent the independent dummy-codes variables in which <1 consumption on average per day represents the reference category; BI or MI measures are the outcome variables; for each pair of independent and dependent variable a separate linear regression model is presented

Model 1: Unadjusted linear regression models; Model 2: Linear regression models adjusted for gender, pubertal status, educational track

B = the estimate of increase in BRI or MI score compared to the reference category

CI=confidence interval

BRI= Behavior Regulation Index; MI=Metacognition Index
